# Ancient Clam Gardens Increased Shellfish Production: Adaptive Strategies from the Past Can Inform Food Security Today

**DOI:** 10.1371/journal.pone.0091235

**Published:** 2014-03-11

**Authors:** Amy S. Groesbeck, Kirsten Rowell, Dana Lepofsky, Anne K. Salomon

**Affiliations:** 1 School of Resource and Environmental Management, Simon Fraser University, Burnaby, British Columbia, Canada; 2 Department of Biology, University of Washington, Seattle, Washington, United States of America; 3 Department of Archaeology, Simon Fraser University, Burnaby, British Columbia, Canada; University of Auckland, New Zealand

## Abstract

Maintaining food production while sustaining productive ecosystems is among the central challenges of our time, yet, it has been for millennia. Ancient clam gardens, intertidal rock-walled terraces constructed by humans during the late Holocene, are thought to have improved the growing conditions for clams. We tested this hypothesis by comparing the beach slope, intertidal height, and biomass and density of bivalves at replicate clam garden and non-walled clam beaches in British Columbia, Canada. We also quantified the variation in growth and survival rates of littleneck clams (*Leukoma staminea*) we experimentally transplanted across these two beach types. We found that clam gardens had significantly shallower slopes than non-walled beaches and greater densities of *L. staminea* and *Saxidomus giganteus,* particularly at smaller size classes. Overall, clam gardens contained 4 times as many butter clams and over twice as many littleneck clams relative to non-walled beaches. As predicted, this relationship varied as a function of intertidal height, whereby clam density and biomass tended to be greater in clam gardens compared to non-walled beaches at relatively higher intertidal heights. Transplanted juvenile *L. staminea* grew 1.7 times faster and smaller size classes were more likely to survive in clam gardens than non-walled beaches, specifically at the top and bottom of beaches. Consequently, we provide strong evidence that ancient clam gardens likely increased clam productivity by altering the slope of soft-sediment beaches, expanding optimal intertidal clam habitat, thereby enhancing growing conditions for clams. These results reveal how ancient shellfish aquaculture practices may have supported food security strategies in the past and provide insight into tools for the conservation, management, and governance of intertidal seascapes today.

## Introduction

Sustaining global food production presents one of the greatest environmental and humanitarian challenges of the 21st century. Given current global population and consumption trajectories, the world's food production must double by 2040 [1], [2] and its footprint must shrink substantially to reduce the degradation of land, water, biodiversity, and climate. Consequently, society will need to develop clever ways to meet demands on terrestrial and marine resources and spaces efficiently, while maintaining ecosystem productivity and resilience. Fortunately, evidence from the past often offers solutions to contemporary quandaries [3], [4]. Here, we provide empirical evidence of an ancient form of mariculture that magnified shellfish production in a limited space, providing practical insights into sustainable marine management techniques which may inform local food security strategies of today.

Humans have been altering, exploiting, and managing marine and terrestrial ecosystems for millennia [5], [6], [7], [8]. Throughout history, human hunting and fishing in coastal ecosystems has caused declines in key species [9], reduced prey size [10], [11], triggered trophic cascades [12], [13], and facilitated ecosystem regime shifts [11]. In other cases, the archaeological record indicates long term sustained yields, with little indication of resource depression [14]. Recent archaeological evidence and oral historical knowledge suggests that First Peoples around the world actively managed and enhanced nearshore ecosystems to maintain and increase productivity [15], [16], [17], [8].

Several ancient environmental engineering and resource management strategies have been documented among coastal indigenous peoples. Enforced harvest size restrictions of forest resources in Fiji [18]; construction and tending of root gardens [16]; transplantation and cultivation of berries to increase yields [19]; and prescribed burns to clear land and magnify plant production [20] along the Northwest Coast provide examples of intentional management of terrestrial coastal resources. Globally, marine examples include stone ponds for fish aquaculture in Polynesia [21]; complex wooden fish weirs in Brittany [22]; and temporal and age-specific harvest restrictions to conserve reef fish in Oceania [15]. Along the northwest coast of North America, marine examples include intertidal stone fish traps at the mouths of salmon bearing streams and intertidal wooden fish weirs [23], [24] in conjunction with size selective fishing practices to enhance salmon productivity [25] and the reduction of predatory sea otters to increase shellfish abundance [7]. These records of direct environmental manipulations, tending, and stewardship practices suggest complex systems of resource management which increased local food security [5], [26], [27].

**Figure 1:**
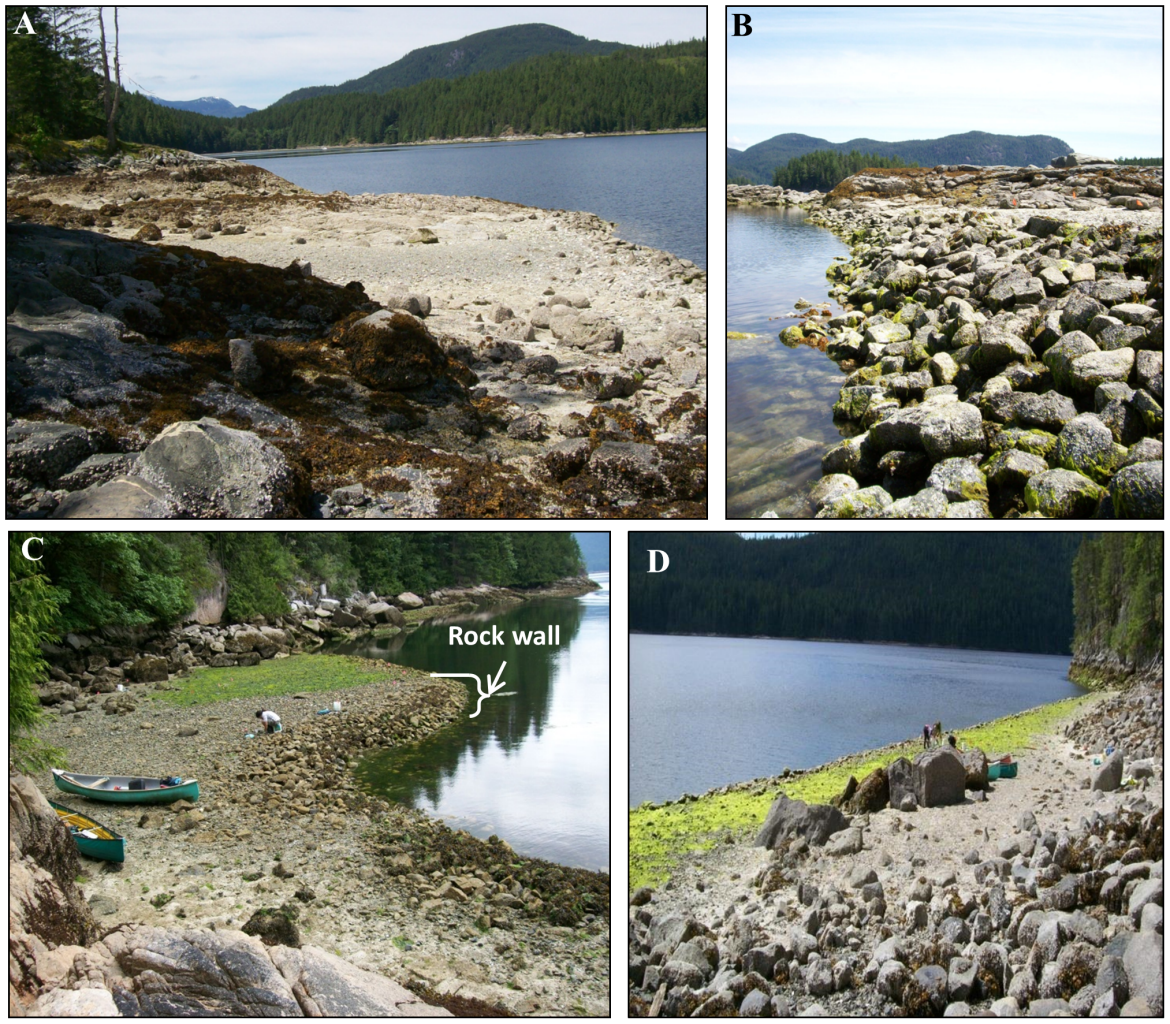
Clam Garden Images. A) Ancient clam gardens on Quadra Island, BC, Canada, are intertidal beach terraces built by humans by constructing B) a rock wall at low tide typically between 0.7–1.3 m above chart datum. C, D) Quadra Island clam gardens range in size and shape but generally create shallow sloping intertidal terraces encompassing tidal heights of 0.9–1.5 m above chart datum.

Researchers have recently turned their attention to ancient clam gardens along the Northwest coast highlighting another example of ancient marine enhancement and management. These human-engineered intertidal terraces, thought to have been constructed in the late Holocene, have been recorded from Alaska through British Columbia (BC) and into Washington State [28]. Made by building rock walls in the low intertidal of soft-sediment clam beaches, clam gardens are thought to stabilize sediments at a specific tidal height, presumably to enhance shellfish productivity [28], [29], [30], [17]. Clam gardens walls exist at the mouths and along the edges of embayments, parallel to coastlines, and vary in shape, length, width, and intertidal height (Fig 1A-D). Although the age of these ancient features is currently unresolved, the immense shell middens associated with clam garden walls suggest the significance of shellfish as a staple food source for Northwest Coast First Nations for at least 5000 years [31]. The combination of the widespread occurrence of clam gardens on the northwest coast, their associated shell middens, and traditional knowledge of clam garden tending passed down in song, story, and practice [32], [16], underscores the importance of these features and suggests that they were constructed to increase clam yields. Knowing how and the extent to which clam gardens boost clam yields may offer insights into contemporary investments in food security for coastal communities.

In this study, we quantified the productivity of ancient clam gardens on Quadra Island, BC with surveys and an *in situ* transplant experiment. We measured how bivalve communities and beach morphology differs between clam gardens and non-walled beaches, and what environmental factors contribute to these differences. Specifically, we ask, do clam gardens have higher clam densities, biomass, and growth rates compared to non-walled beaches? And if so, what physical characteristics best explain these differences?

## Methods

### Study Area

**Figure 2:**
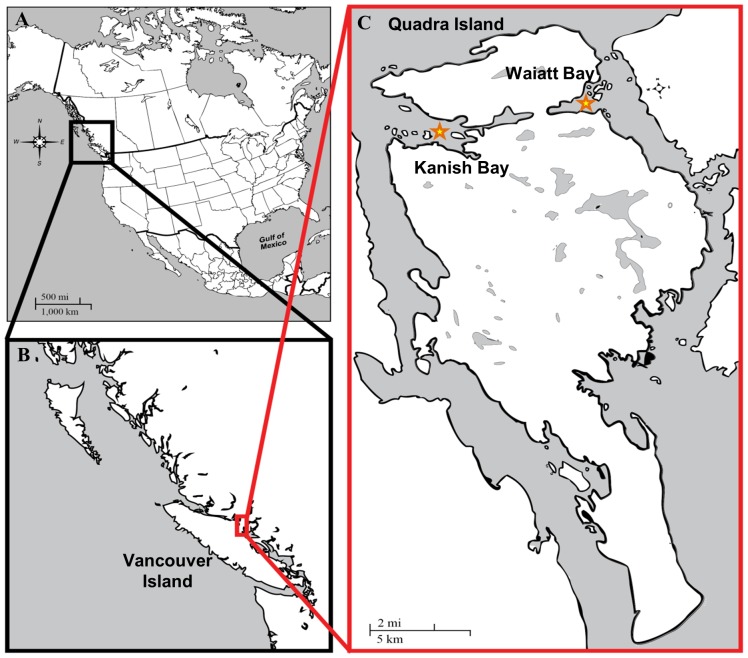
Map of Study Area. This research was conducted on A) the west coast of British Columbia, Canada, in the Inside Passage between B) Vancouver Island and the mainland on the northern end of C) Quadra Island, in Kanish Bay (West, starred) and Waiatt Bay (East, starred).

We conducted our research on northern Quadra Island in British Columbia (BC), Canada, where an exceptionally high density of clam gardens have been documented [30] in Kanish (n =  45 clam gardens) and Waiatt Bays (n =  49 clam gardens) (Fig 2). Quadra Island has an abundance of archaeological sites found throughout the landscape, with shell middens representing both permanent settlements and short term camps. Today, the northern part of the island falls within the traditional territories of the Northern Coast Salish and the Southern Kwakwaka’wakw (now Laich-kwil-tach) First Nations. Some of the descendants of the ancient settlements live in nearby Indian Reserves and town centers. Presently, Kanish and Waiatt Bays are only sparsely settled and bordered by second growth forests and active wood lots. The bays are popular recreation areas and anchorages, encompassing two provincial parks and an active scallop farm. Clam digging, once a mainstay of the dense human population in the bays, is now only conducted recreationally and sporadically.

The soft sediment, low wave energy, intertidal shores of Kanish and Waiatt Bays foster bivalve communities of *Leukoma staminea* (native littleneck) and *Saxidomus giganteus* (butter clams), both ecologically, economically, and culturally important clam species. Other common bivalves include the native *Macoma spp*. (macoma clams), *Clinocardium nuttallii* (heart cockles), *Tresus nuttallii* and *Tresus capax* (horse clams), and the non-native *Venerupis philippinarum* (Japanese littlenecks), and *Mya arenaria* (eastern softshell clams).

### Field Surveys

We located 5 non-walled clam beaches in each bay that we deemed appropriate controls for our study. We then chose at least 5 clam gardens in each bay as treatments for comparison. The total number of non-walled beaches (n = 10) and clam gardens (n = 11) surveyed for physical and biological comparison was constrained by the number of low tide days available during the spring and summer field season of 2011.

We characterized beach slope at 11 clam gardens and 10 non-walled beaches to quantify the physical differences in intertidal clam habitat between clam gardens and non-walled clam beaches. At each site, we established a vertical transect, perpendicular to shore, with 15 randomly stratified stations. The tidal height of each station was quantified using a total station or laser level in meters above Canada chart datum, lowest low water large tide (LLWLT). Transects began at the highest intertidal height at which clams were found in test pits. In clam gardens, the bottom tidal height of each transect was anchored by the landward edge of the human-made rock wall. At non-walled clam beaches, the bottom tidal height of each transect was anchored at ∼1.0 m above chart datum, the mean tidal height of the landward edge of the clam garden rock-walls.

**Figure 3:**
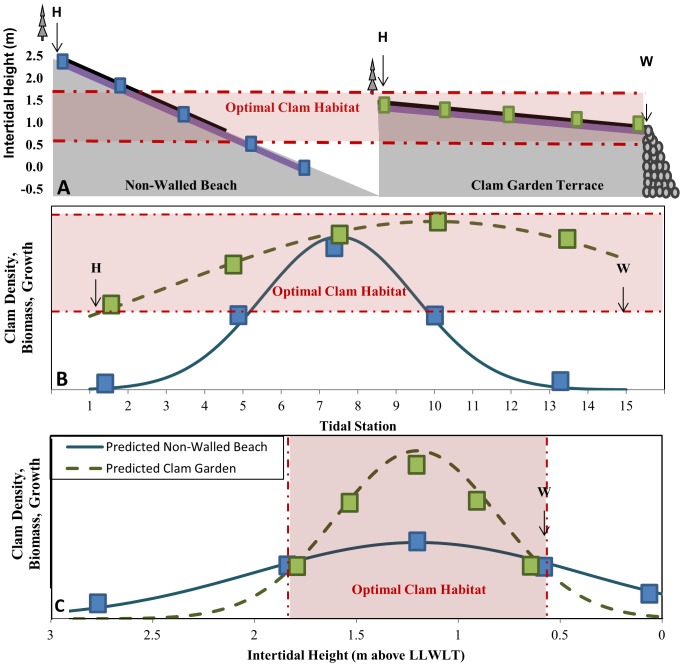
Study Design and Predictions. A) We surveyed clams across a vertical transect (black line) from the top of clam habitat (H) to ∼1.0 m in non-walled beaches (left) and to the edge of the rock wall (W) in clam gardens (right). We then transplanted clams in mesh bags at 5 evenly stratified tidal stations (blue and green colored squares) across a vertical transect (purple line) from the top of clam habitat (H) to ∼0 m in 5 Non-Walled Beaches (left) and to the edge of the rock wall (W) in 6 clam gardens (right). B) Hypothesis 1: predicted clam productivity as a function of tidal station. Tidal station 1  =  top of clam habitat, tidal station 15  =  top of clam garden wall in clam gardens or ∼1.0 m tidal height in non-walled beaches. C) Hypothesis 2: predicted clam productivity as a function of intertidal height.

To test for differences in bivalve composition, density, size, and biomass between the 11 clam gardens and 10 non-walled clam beaches, we dug sample units (25×25×30 cm  =  0.018 m^3^) at the 15 tidal stations along the vertical transect (Fig 3a). Live clams were identified to species, and their wet weight, maximum longitudinal valve length, and width were measured.

### Clam Transplant Experiment

To test if clam gardens increase the growth rates of *L. staminea*, we conducted a transplant experiment across six clam gardens and five non-walled clam beaches during the growing season from May to October 2011 [33]. Clams 11–34 mm in length were collected from Waiatt and Kanish Bay, labelled with two uniquely numbered vinyl tags, measured to the nearest 0.1 mm and weighed to the nearest 1.0 gram. Fifteen individuals, representing the range of sizes collected, were placed inside a 34 cm×24 cm Vexar mesh bag. Five bags of *L. staminea* were evenly spaced at five tidal stations along a single vertical transect, perpendicular to shore, from the top of clam habitat to ∼0 m intertidal height at non-walled beaches and at the edge of rock wall in clam gardens (Fig 3a). Each bag was buried approximately 10 cm below the surface, based on our natural history observations for clams of this size and recommendations from a contemporary aquaculture facility. Each transplant bag was then secured with a flagged and labelled rebar stake. Transplanted clams were left *in situ* for 160 days. Upon retrieving the transplanted clams, max lengths, widths, and weights were recorded. We noted all losses, mortalities, and evidence of predation.

We chose *L. staminea* for our experiment because they are a biologically and culturally significant species in the area, and they possess distinct annuli, which have been verified by the annual temperature-driven δ^18^O signature observed between annuli (K Rowell, pers. comm.). For the same reasons *L. staminea* will also be used in a companion archeological study comparing ancient clam growth rates through deep time, both before and after clam garden construction.

### Predictions

By constructing a rock-walled terrace in the intertidal, we predicted that clam gardens expand optimal clam habitat by altering the slope of the beach and thus increasing clam habitat at the intertidal height at which clams grow and survive best (Fig 3a). In response to increased habitat and enhanced conditions we predicted that clam density, biomass, growth, and survival would increase at the first and last tidal stations (Fig 3b), i.e., the extreme high and low of intertidal heights (Fig 3c), in clam gardens compared to non-walled beaches. We also predicted that Gaussian models of optimal clam productivity as a function of intertidal height would peak at the same intertidal height in both clam gardens and non-walled beaches, but that the magnitude of productivity would be greater (i.e. due to increased water retention, differences in sediment composition, or both) and the variance smaller (i.e. due to the reduction in beach slope) in clam gardens relative to non-walled beaches (Fig 3c).

We selected clam garden sites based on the presence of a complete rock wall spanning an embayment. Non-walled control beaches were beaches that lacked a rock wall but encompassed clam habitat, specifically intertidal soft sediment and the full array of clam species observed in the area. When comparing non-walled beaches and clam gardens, we assume that contemporary non-walled beaches are representative of the pre-construction state of clam garden sites.

### Data Analysis

#### Physical Site Characteristics

To test for an effect of *beach type* (i.e., clam garden [n = 11] vs. non-walled beach [n = 10]) and an effect of *bay* (Kanish vs. Waiatt Bay) on beach slope, we used a general linear model (GLM). We used the same strategy to examine differences in heights of clam garden walls and mean heights of garden terraces between bays. Models of Slope, Wall Top Height, and Terrace Height were fit with a Restricted Maximum Likelihood (REML), a Gaussian error distribution, and identity link function using the lme function in the nlme package [34].

#### Field Surveys

To test for differences in clam density and biomass between clam gardens (n = 11) and non-walled clam beaches (n = 10), we constructed general linear mixed effects models (GLMMs) where *beach type* was treated as a fixed effect and *site* was treated as a random effect. These models were constructed for the three most dominant species (*L. staminea*, *S. giganteus*, and *Macoma* spp.) and total clams. To test for differences in *L. staminea* density among different size classes, we ran the same models described above on five size classes of clam binned into categories based on 12 mm increments. Bin size was determined by the size range of the surveyed clams and number of clams in each bin that would yield sample sizes large enough for sufficient statistical power. Differences in clam density and biomass between clam gardens (n = 11) and non-walled clam beaches (n = 10) as a function of tidal station in both Kanish and Waiatt Bay were assessed using the same GLMMs as described above, with the additional fixed effects of *bay* and the interaction of beach type*tidal station. *Beach type*, *bay,* and type*tidal station were specifically chosen as treatments to be tested, beach *type* to detect a clam garden effect, *bay* to detect an effect of oceanographic context, and type*tidal station to detect our predicted across-beach effect of tidal station in clam gardens and non-walled beaches (Fig 3b). Clam biomass models were fit with a REML, a Gaussian error distribution, and identity link function using the lme function in the nlme package [34]. Clam density models were fit with Laplace Approximation, a Poisson error distribution, and log link function using the lmer function and lme4 package [35]. All GLM and GLMM modelling was conducted in R [36].

#### Optimal Clam Habitat Models

To assess if and how clam garden engineering altered intertidal height and optimal growing conditions for clams, we modeled the relationship between intertidal height and a) density and biomass of surveyed *L. staminea* and b) survivorship and growth of transplanted *L. staminea* in clam gardens and non-walled beaches in both bays, by fitting Gaussian models (Eq.1) to each metric of clam productivity (*y*) as a function of intertidal height where*:*

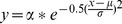
(Eq.1)α (curve height) describes the magnitude of clam productivity (biomass, density, or growth rate), µ (curve mean) is the intertidal height at which productivity is greatest, and *σ* (curve width) describes the standard deviation in clam productivity. We then compared fitted model parameters across clam gardens and non-walled beaches in both bays based on our predictions (Fig 3).

#### Experimental Transplants

We tested for differences in survival and growth rates of *L. staminea* transplanted in clam gardens (n = 6) and non-walled clam beaches (n = 5) in Waiatt Bay, using generalized GLMMs where *beach type* was a fixed effect and *site* was a random effect. To test for differences in *L. staminea* growth and survival across tidal stations within clam gardens and non-walled beaches, we constructed the same GLMMs as above, with *beach type*, *bay* and beach type*tidal station as fixed effects. Growth rates models were fit with REML, a Gaussian error distribution, and identity link function using the lme function in the nlme package [34]. Survivorship models were fit with a Laplace Approximation, a binomial error distribution, and logit link function using the lmer function and lme4 package [35]. Survivorship and growth were compared among different juvenile clam size classes using the same models described above on three size classes of transplanted *L. staminea* binned by 5mm increments. Bin size was determined by the size of the transplanted clams and number of clams in each bin that would yield sample sizes large enough for sufficient statistical power.

#### Ethics Statement

All field work was conducted with the following permits: a Contaminated Shellfish Collection for Scientific Purpose Permit from Department of Fisheries and Oceans Canada, a Park Use Permit from BC Parks, an Animal Care Protocol Exemption from Canadian Council on Animal Care for working with invertebrates, and permission from Laichwiltach Treaty Society. The individual pictured in this manuscript (Figure 1C and Striking Image) has given written informed consent (as outlined in PLOS consent form) to publish their likeness. Our study did not involve endangered or protected species. All necessary permits were obtained for the described study, which complied with all relevant regulations.

## Results

### Physical Characteristics of Beach Types

**Figure 4:**
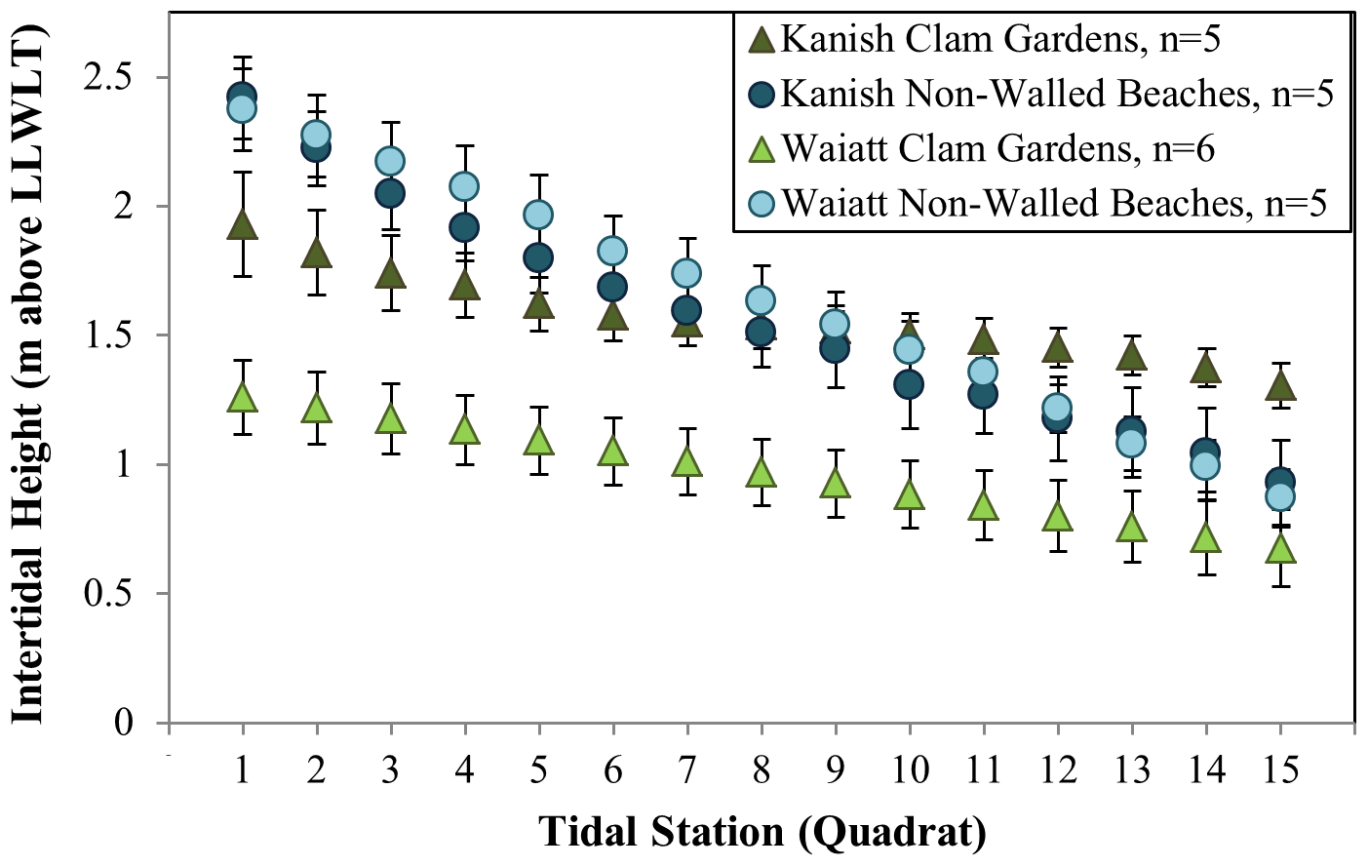
Site Descriptions: Intertidal Height by Tidal Station. Intertidal height (mean +/–SE) and relative slope (▵y/▵x) from top of clam habitat (tidal station  =  1) to top of rock wall feature in clam gardens and ∼1.0 m above LLWLT in non-walled beaches (tidal station  =  15).

On Quadra Island, BC, clam garden terrace heights varied between bays, but their slopes were consistently shallower than unaltered beaches (Fig 4, S2 a, Table S1, F_(1,19)_ = 6.914, *p = *0.017). On average, mean intertidal heights of clam garden terraces in Waiatt Bay were significantly lower than those in Kanish Bay (Fig 4, S1, S2 a, Table S1, F_(1,9)_ = 15.848, *p = *0.003). In Waiatt Bay, clam garden terraces were located on average at 0.97 m (+/– 0.31 SE) above chart datum, while the tops of rock wall features averaged 0.68 m (+/– 0.36 SE) in intertidal height (Fig 4, S1). In contrast, Kanish clam garden terraces were located on average at 1.57 m (+/–0.21 SE) above chart datum and the tops of rock wall features averaged 1.3 m (+/–0.19 SE) in intertidal height (Table S2). Non-walled beach slopes and mean intertidal heights did not differ between bays (Fig 4, S2 a,b).

We observed greater variation in the intertidal height of clam gardens in Waiatt Bay. There, clam garden terraces were located between 0.53–1.45 m above chart datum, with four of the six clam gardens having mean terrace heights between 0.78–1.16 m, and two outliers having mean terrace heights of 0.53 m and 1.45 m respectively (Fig 4, S1).

### Field Surveys

**Figure 5:**
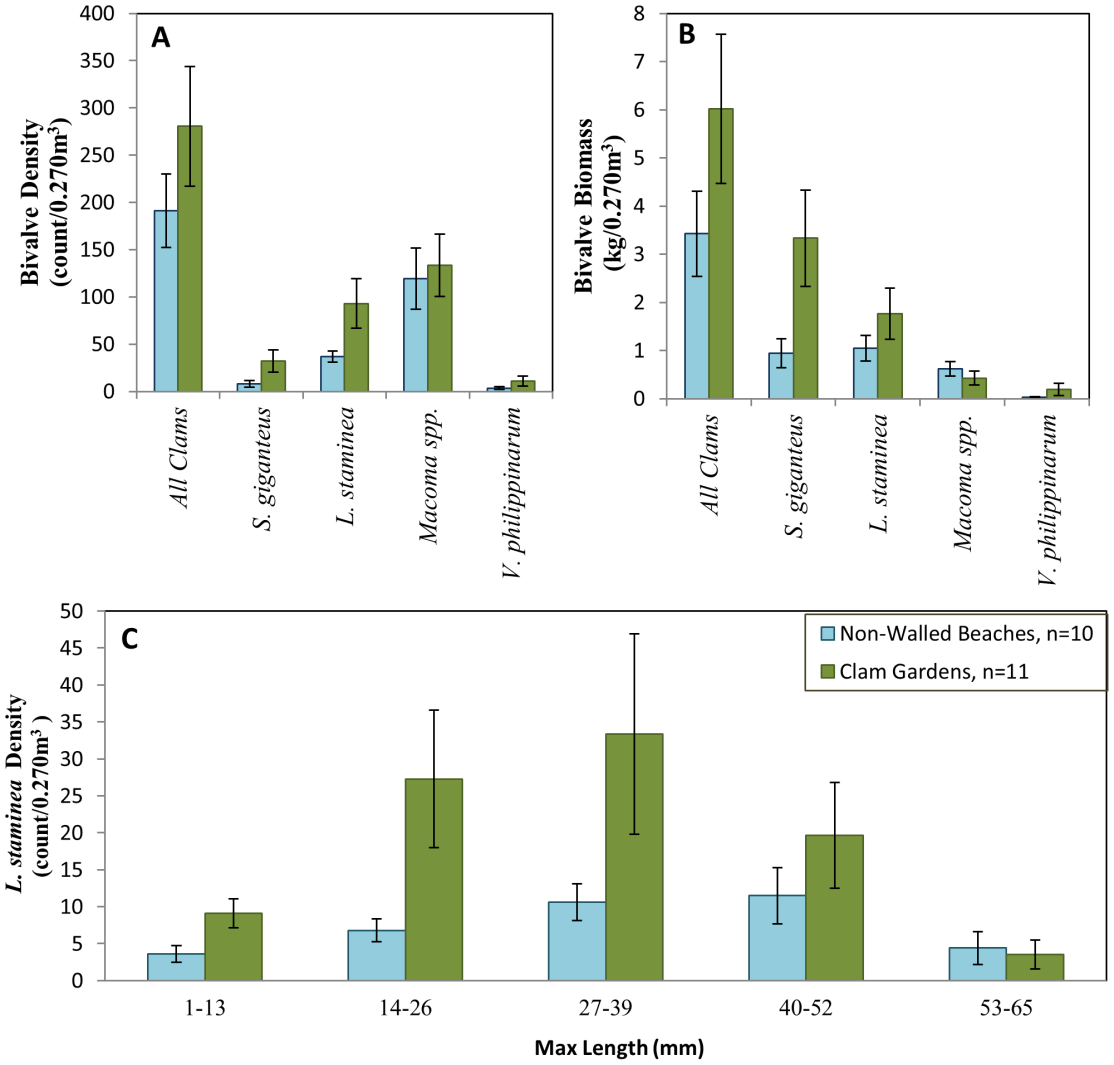
Survey: Bivalve Biomass and Density. B) Density (count/0.270 m3 +/–SE) and C) Biomass (kg/0.270 m3 +/–SE) of four most abundant bivalve species; A) *L. staminea* density (count/0.270 m3 +/–SE) of 5 size classes in Clam Gardens and Non-Walled Beaches.

*L. staminea* and *S. giganteus* dominated the subsistence species bivalve community in clam gardens and non-walled beaches, both in density and biomass, (Fig 5a,b). We detected significantly higher densities of *L. staminea,* in clam gardens (93 +/–26se #/0.270 m^3^) than in non-walled beaches (37 +/–6 se #/0.270 m^3^, *p* = 0.03, Fig 5a, Table 1, S2). Differences were more pronounced at smaller size classes (Fig 5c, Table 1, S2). Densities of *S. giganteus* were also significantly greater in clam gardens (32 +/–12 se #/0.270 m^3^) compared to non-walled beaches (8 +/–4 se #/0.270 m^3^)(Fig 5a, Table 1,S2), and these clams tended to be larger in clam gardens, yielding on average higher biomass (3.3 +/–1.0se kg/0.270 ^3^) compared to non-walled beaches (0.94 +/–0.3se kg/0.270 m^3^)(Fig 5b, Table 1, S2). The density and biomass of other documented bivalve species did not differ as a function of beach type (Table 1). Surveyed clam biomass of all species combined was nearly double within clam gardens (6.02 +/–1.55se kg/0.270 m^3^) compared to non-walled beaches (3.43 +/– 0.88se kg/0.270 m^3^ ^3^) (Fig 5b), however due to the variation among sites within beach type, overall bivalve biomass did not significantly differ between beach types (Fig 5b, Table 1, S2).

**Table 1:** Clam garden effect on density and biomass.

	Fixed Effect		Random Effect	
Response variable	Beach Type		Site (Beach Type)	
Density	*z*	***p***	**Variance**	**StdDev**
*L. staminea* (All)	–**2.24**	0.03*	0.60	0.78
*S. giganteus*	–**2.25**	.03*	2.38	1.54
*V. philippinarum*	–0.69	0.49	4.66	2.16
*Macoma spp.*	0.05	0.96	1.51	1.23
TOTAL clam	–1.01	0.32	0.53	0.73
*L. staminea* (1–13 mm)	–**2.49**	0.01*	0.61	0.78
*L. staminea* (14–26 mm)	–**2.76**	<0.01*	0.77	0.87
*L. staminea* (27–39 mm)	–**2.11**	0.04*	0.73	0.86
*L. staminea* (40–52 mm)	–1.06	0.29	1.12	1.06
*L. staminea* (53–65 mm)	0.18	0.86	3.70	1.92
Biomass	*t*	***p***	**Residual**	
*L. staminea*	–1.16	0.26	0.10	
*S. giganteus*	–1.77	0.09	0.24	
*V. philippinarum*	–1.23	0.24	0.03	
* Macoma spp.*	1.41	0.17	0.04	
TOTAL clam	–0.20	0.85	0.33	

The effect of clam gardens (Beach Type) on density and biomass (per survey transect, 0.027 m^3^) of *L. staminea* (littleneck clam), *S. giganteus* (butter clam), *V. philippinarum* (Japanese littleneck clam), *Macoma* spp (macoma clams) and total clams. * designates significant p-values (p≤0.05).

**Figure 6:**
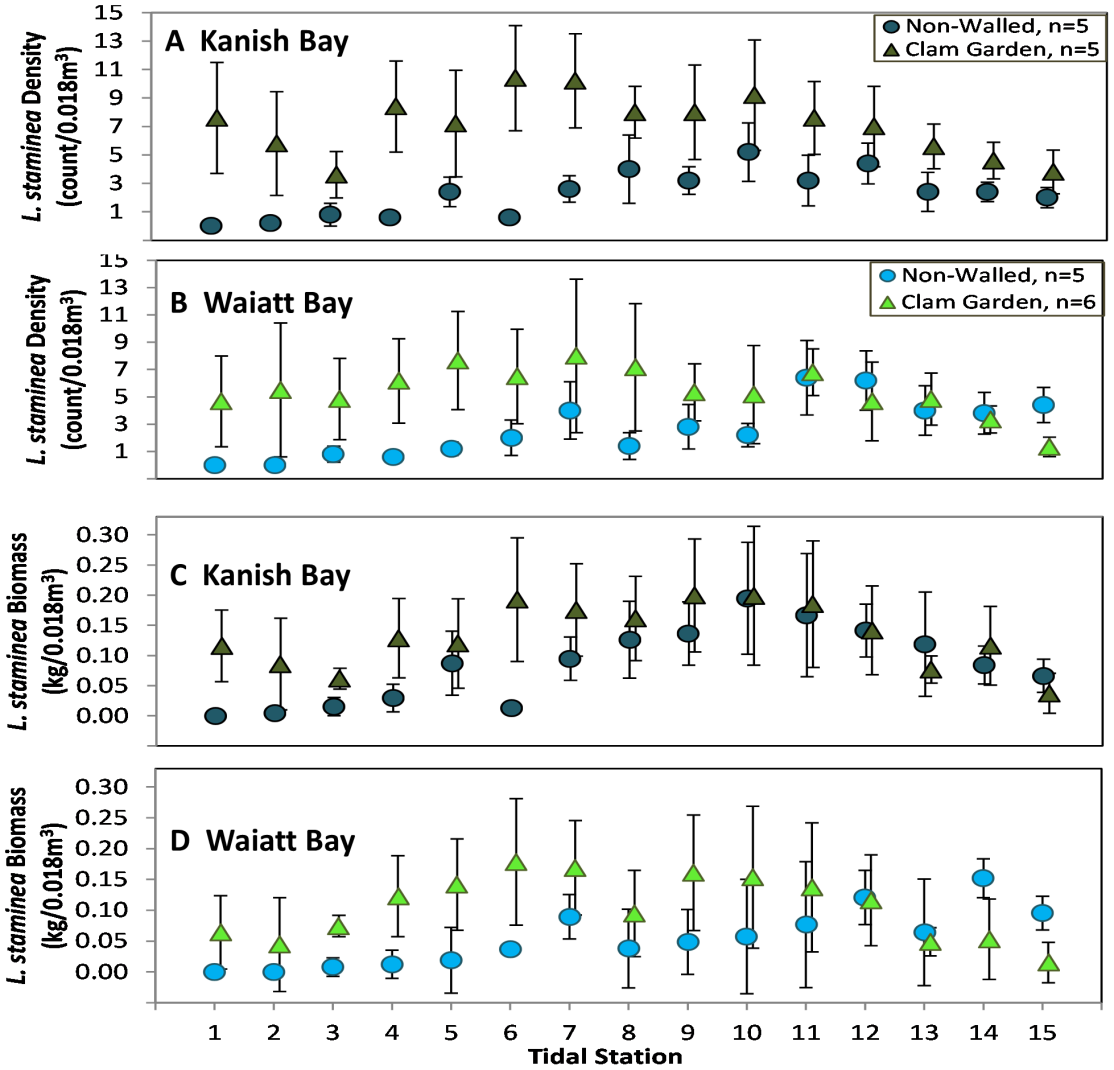
Survey*: L. staminea* Biomass and Density by Tidal Station. Surveyed A&B) density (count/0.018m3 +/– SE) and C&D) biomass (kg/0.018m3 +/– SE) and of *L. staminea* as a function of bay (Kanish or Waiatt), site type (Clam Garden or Non-Walled Beach), and tidal station (1 = top of clam habitat, 15 =  top of clam garden wall or ∼0.75 m intertidal height in non-walled beaches).

By examining clam density and biomass as a function of tidal station and beach type in both bays, we found that *L. staminea* and *S. giganteus* densities and biomass were significantly greater in clam gardens than non-walled beaches and as predicted (Fig 3b), this relationship varied as a function of tidal station (Fig 6). This is reflected by the significant interaction terms in Table 2. Specifically, clam densities and biomass tended to be higher at the first 6–7 tidal stations. The effect of tidal station position was highly significant for total clam densities and densities of *L. staminea* (all sizes, 14–26 mm, 27–39 mm) (Fig 6, Table 2), *S. giganteus*, and *V. philippinarum* (Table 2). The density and biomass of the invasive *V. philippinarum* varied significantly as a function of tidal station and was significantly higher in Kanish Bay (Table 2).

**Table 2:** Effects of clam garden treatment and oceanographic context.

	Fixed Effect								Random Effect	
Response Variable	Beach Type		Bay		Tidal Station		Beach Type x Tidal Station		Site	
**Density**	***z***	***p***	***z***	***p***	***z***	***p***	***z***	***p***	**Var**	**SD**
** ** ***L. staminea*** ** (All)**	–**5.78**	<0.01*	–0.21	0.83	–3.47	<0.01*	**10.34**	<0.01*	0.60	0.77
** ** ***S. giganteus***	–**4.72**	<0.01*	–0.52	0.96	–4.29	<0.01*	**7.14**	<0.01*	2.38	1.54
*** V. philippinarum***	–0.32	0.75	–0.40	0.69	–3.47	<0.01*	–1.16	0.25	4.61	2.15
** Macoma**	–**2.69**	0.01*	–0.87	0.39	0.12	0.91	**15.97**	<0.01*	1.46	1.21
** TOTAL clam**	–**4.50**	<0.01*	–0.55	0.58	–4.03	<0.01*	**18.21**	<0.01*	0.53	0.73
***L. staminea*** ** (1**–**13 mm)**	–**3.02**	<0.01*	0.59	0.56	0.90	0.37	**1.93**	0.05*	0.62	0.79
***L. staminea*** ** (14**–**26 mm)**	–**5.63**	<0.01*	0.32	0.75	–3.62	<0.01*	**6.17**	<0.01*	0.77	0.88
***L. staminea*** ** (27**–**39 mm)**	–**4.96**	<0.01*	1.48	0.14	–3.18	<0.01*	**6.13**	<0.01*	0.67	0.82
***L. staminea*** ** (40**–**52 mm)**	–**2.74**	<0.01*	–1.72	0.09	–0.06	0.95	**3.57**	<0.01*	0.92	0.96
***L. staminea*** ** (53**–**65 mm)**	–1.19	0.23	0.94	0.35	0.77	0.44	**2.81**	<0.01*	3.59	1.90
**Biomass**	***t***	***p***	***t***	***p***	***t***	***p***	***t***	***p***	**Residual**	
** ** ***L. staminea***	–**2.77**	0.01*	–0.71	0.49	–0.59	**0.55**	**3.92**	<0.01*	0.10	
** ** ***S. giganteus***	–**2.59**	0.02*	1.05	0.31	–0.89	**0.37**	**2.21**	0.03*	0.24	
*** V. philippinarum***	–**2.16**	0.04*	–1.45	0.17	–3.03	<0.01*	1.83	0.07	0.03	
*** Macoma spp.***	–0.92	0.37	–1.56	0.14	–0.62	0.53	4.14	0.00	0.04	
** TOTAL clam**	–0.83	0.42	–0.56	0.58	0.60	0.55	1.36	0.17	0.33	

The effects of clam gardens (Beach Type), oceanographic context (Waiatt Bay vs. Kanish Bay), and Tidal Station on the biomass and density of surveyed *L. staminea*, *S. giganteus*, *V. philippinarum*, *Macoma spp*, and total clams (per survey transect, 0.027 m^3^). * designates significant p-values (p≤0.05).

### Effect of Beach Engineering on Optimal Clam Habitat

**Figure 7:**
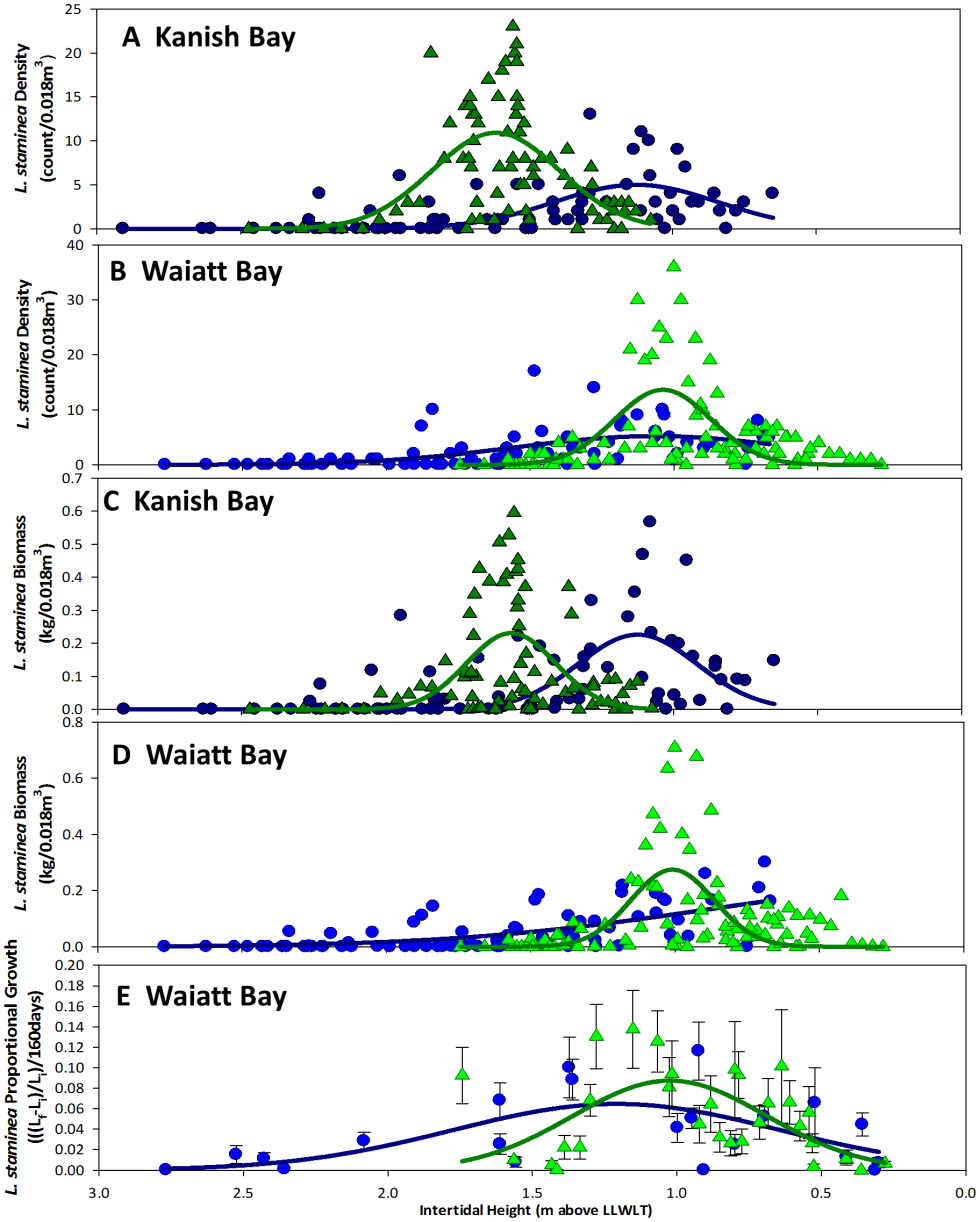
Survey & Experiment: *L. staminea* Biomass, Density and Growth by Intertidal Height vs. Model Predictions. Actual and predicted A&B) density (count/0.018 m3 +/− SE) C&D) biomass (kg/0.018 m3 +/− SE) and E) growth (mean +/−SE) of surveyed (A-D) and transplanted (E) *L. staminea* as a function of intertidal height (m above LLWLT) in clam gardens (green triangles) and non-walled beaches (blue circles) in Kanish and Waiatt bays, British Columbia, Canada.

As predicted (Fig 3c), the magnitude of *L. staminea* productivity (*a*) in terms of density, biomass, and growth, was higher in clam gardens than non-walled beaches, and the standard deviation (*σ)* was lower (Fig 7a-d, Table 3). Contrary to expectations, the intertidal height at which little neck clams reach their maximum density, biomass, and growth (µ) was consistently higher in clam gardens than non-walled beaches in Kanish Bay (Fig 7, S4, Table 3), suggesting that optimal habitat shifted ∼0.5 m higher up the beach in clam gardens in Kanish. In Waiatt, maximum *L. staminea* productivity (µ) did not differ substantially between beach types, and biomass did not conform to a Gaussian relationship within non-walled beaches.

**Table 3:** Gaussian models: effects of clam garden treatment and intertidal height.

Predictive Gaussian Curves, 3 parameter: *y* = a*exp(–0.5*((x-µ)/σ)^2^)							
Method	Bay	Type	Response	a (height)	µ (mean)	σ (variance)	R^2^
Survey	Kanish	NW	*L.s.* Density	5.000	1.126	0.286	0.327
Survey	Kanish	CG	*L.s.* Density	10.913	1.613	0.222	0.380
Survey	Waiatt	NW	*L.s.* Density	5.219	1.065	0.487	0.291
Survey	Waiatt	CG	*L.s.* Density	13.640	1.038	0.167	0.323
Survey	Kanish	NW	*L.s.* Biomass	0.226	1.125	0.207	0.315
Survey	Kanish	CG	*L.s.* Biomass	0.231	1.564	0.155	0.258
Survey	Waiatt	NW	*L.s.* Biomass	0.216	0.015	0.882	0.398
Survey	Waiatt	CG	*L.s.* Biomass	0.274	1.009	0.144	0.322
Transplant	Waiatt	NW	*L.s.* Growth	0.065	1.209	0.566	0.362
Transplant	Waiatt	CG	*L.s.* Growth	0.088	1.027	0.331	0.283

Parameters for the modeled responses of biomass (kg/0.018 m^3^ +/− SE), density (count/0.018 m^3^ +/− SE), and growth (mean +/−SE) of surveyed and transplanted *L. staminea* (*L.s.*) as a function of intertidal height. Each response was predicted by modeling a Gaussian curve to the data, *y*  =  a*exp(−0.5*((*x*−µ)/*σ*)^2^) (Eq. 1), where *y* = response, *x* = intertidal height, *a* = height, µ = mean, and *σ* = standard deviation.

### Experimental Transplants

**Figure 8:**
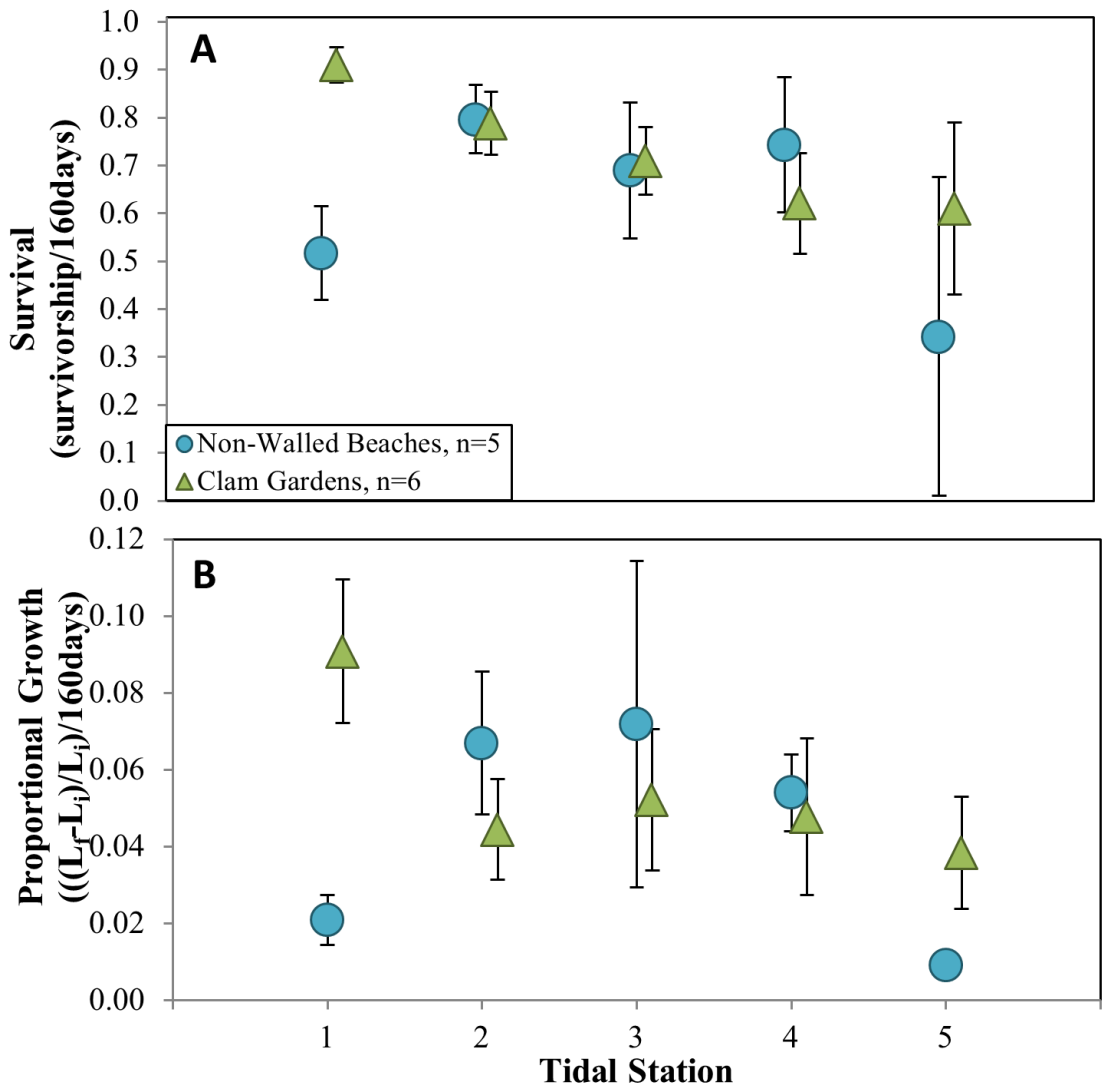
Experiment: *L. staminea* Growth and Survival by Tidal Station. A) Survival and B) growth rates (+/−SE) of transplanted *L. staminea* as a function of tidal station. Tidal station 1 was anchored at top of clam habitat and tidal station 5 was located at the top of the rock wall feature in clam gardens, and at ∼0 m below LLW in non-walled beaches.

Transplanted *L. staminea* grew significantly faster in clam gardens than non-walled beaches, and this effect varied as a function of tidal station (Fig 8, S3, Table 4,S3). Clams grew proportionally faster at tidal station extremes (the first and last tidal station) in clam gardens compared to non-walled beaches. In line with our expectations, the overall magnitude of growth rates as a function of tidal height was higher in clam gardens than non-walled beaches (Fig7e, S4, Table 3). Size appears to be a major predictor of survivorship - small size classes of *L. staminea* (11−16 and 17−22 mm) were more likely to survive in clam gardens than non-walled beaches, although clam garden habitat did not appear to effect survivorship when all size classes were pooled. This size dependent effect varied as a function of tidal station (Table 4).

**Table 4:** Experimental effects of clam garden treatment and tidal station.

	Fixed Effect								
Response Variable	Beach Type			Tidal Station			Beach Type x Tidal Station		
**Growth Rates**	***df***	***F***	***p***	***df***	***F***	***p***	***df***	***F***	***p***
Growth rate (All sizes)	1,9	8.79	0.02*	1,666	22.05	<0.01*	1,666	8.62	<0.01*
Growth rate (11−16 mm)	1,9	1.57	0.242	1,87	3.90	0.05*	1,87	0.38	0.54
Growth rate (17−22 mm)	1,9	<0.01	0.980	1,197	0.13	0.720	1,197	0.08	0.78
Growth rate (23−28 mm)	1,9	0.23	0.646	1,125	1.30	0.256	1,125	0.01	0.92
**Survival**	***SE***	**z**	***p***	***SE***	**z**	***p***	***SE***	**z**	***p***
Survivorship (All sizes)	1.53	−0.98	0.33	0.31	−1.28	0.20	0.47	0.77	0.44
Survivorship (11−16 mm)	0.95	−1.92	0.05*	0.14	−2.66	<0.01*	0.27	1.19	0.23
Survivorship (17−22 mm)	0.81	−2.50	0.01*	0.14	−4.15	<0.01*	0.20	2.73	<0.01*
Survivorship (23−28 mm)	1.18	−0.01	0.99	−0.48	0.20	0.02*	0.32	0.07	0.95

The effects of clam gardens (Beach Type) and tidal station on the growth and survivorship of transplanted *L. staminea*. * designates significant p-values (p≤0.05).

## Discussion

Strong evidence from both our surveys and experimental transplants, suggests that ancient clam gardens increased clam productivity. By altering the slope of soft-sediment beaches (Fig 4), these human-made, intertidal terraces expanded the optimal intertidal habitat and enhanced growing conditions for clams. Specifically, we detected significantly greater densities of *S. giganteus* and *L. staminea* in clam gardens compared to non-walled beaches, particularly among smaller size classes of pre-reproductive clams (Fig 5). Overall, clam gardens contained 4 times as many butter clams (*S. giganteus*) and over twice as many little neck clams (*L. staminea*) relative to non-walled beaches (Fig S2). As predicted, the magnitude of this relationship varied as a function of intertidal height, whereby clam density and biomass was enhanced in clam gardens compared to non-walled beaches at the top and bottom of the beach, the areas where clam gardens extend optimal clam habitat (Fig 6,8). The pattern of increased clam productivity by clam gardens appears to be driven by the modification of intertidal height (Fig 7) and is supported by our experimental results indicating higher *L. staminea* growth rates within clam gardens (Fig 7e,8). Even though clam gardens on Quadra Island have not been actively tended or managed for decades, we detected significant signals of enhanced shellfish production across these engineered beaches simply due to their modified slopes. Furthermore, elevated clam densities, biomass, and growth rates at equivalent intertidal heights in clam gardens compared to non-walled beaches suggests that additional mechanisms, in addition to tidal height modification, appear to be magnifying secondary production in these clam terraces.

### Mechanisms Enhancing Productivity in Clam Gardens

In addition to altering beach slope and thereby extending the area of intertidal habitat at the optimum tidal height for clam survival and growth, clam gardens terraces may have enhanced clam productivity in multiple ways. For example, over the diurnal tidal exchange, we observed increased water retention over clam gardens relative to natural sloping non-walled beaches. Water retention may increase the opportunity and success of larval clam recruitment and survivorship in a clam garden. In fact, a Hul’qumi’num First Nation reported that clam gardens were a way to “trap the seeds and keep them here” [27]. Low water velocities create optimal conditions for larval settlement and recruitment [37]. Water retained over shallow sloping clam gardens could increase in temperature in the late spring and summer months thereby increasing phytoplankton growth rates [38] and fuelling secondary production[39], [40]. Furthermore, increased water temperatures in temperate intertidal systems are known to enhance bivalve growth rates [41], [42] and trigger bivalve spawning events [33].

By intentionally modifying substrate, reducing density dependence, and excluding competitors and predators in clam garden terraces, indigenous people may have enhanced clam productivity in clam gardens even further. On Quadra Island, we observed that clam garden substrate tended to be higher in gravel and shell hash content compared to non-walled beach substrate, which tended to have more fine sediments. Similar observations have been reported elsewhere [23], [28] and *L. staminea,* are commonly found on natural beaches in course sand or fine gravel mixed with mud, stones, or shells [43], [44]. According to a Heiltsuk First Nation knowledge holder; "We put gravel in the garden to increase the number of clams" (D Wilson, pers. comm.). Increasing the gravel content in clam gardens may have created larger interstitial spaces in the substrate which is likely important for porewater flow and reduced fine silts and clays that are known to smother newly settled *L. staminea* larvae [43]. The act of aerating beach sediments by rolling rocks, or “turning over beaches” is also commonly reported [32], [5], [27] and aims to reduce anoxic conditions that can reduce productivity. In addition, Hul’qumi’num First Nation knowledge holders report returning crushed and whole clam shells to clam gardens as a management practice [27] and adult bivalve shell has been shown to offer an important settling cue for shellfish like oysters and clams [45], [46]. In fact, it has been demonstrated that gravel and shell increase clam settlement and survival [47]. Finally, Turner [5] reports that clam gardens were “thinned,” reducing densities of large adult clams via harvest, giving smaller clams the space and resources to grow, and thus increasing overall yields [48]. We also hypothesize that predators such as sea stars (*Pycnopodia helianthoides*, *Pisaster brevispinus*.), large crabs (*Metacarcinus magister)* and mammalian coastal predators (river otters, sea otters), may have been intentionally excluded from these gardens to decrease both direct predator mortality and negative non-lethal predator effects on clam productivity [49], [50].

In addition to these ecological factors, several key social factors, including systems of tenure and control, may have equally enhanced and maintained the productivity of clam gardens. Indigenous peoples of the northwest coast had territorial governance systems and complex protocols that delineated access rights to the land and sea [51], [5], [52]. Clam gardens, like seaweed picking areas, root gardens, and fish traps, would have been embedded in these traditional systems of marine governance and tenure. Among the Heiltsuk First Nation, families owned and tended productive clam beaches (D Wilson, pers. comm.). Building and maintaining clam gardens were intentional acts, clearly showing cultural investment. Territorial access rights, via family-based proprietorship, established a governance system over common pool fisheries resources that likely conferred resilience to societies on the Northwest coast for millennia [52], [8]. Similarly, empirical evidence from contemporary fisheries management highlights the importance of designating access rights to enhance resource sustainability [53], [54].

### Assumptions

Documenting ancient resource management within contemporary landscapes presents a challenge for various reasons, one being the identification of adequate controls. Comparisons of areas with and without archaeological features are complicated by the uncertainty in why suitable land and seascapes were not modified. In this study, we assumed naturally shallow sloping non-walled clam beaches are appropriate clam garden controls; however a variety of alternative and non-mutually exclusive hypotheses could explain why some beaches were left unmodified. For example, non-walled beaches could have been of poor quality habitat, used for other purposes, owned by other title holders or may have been at an inappropriate tidal height due to past sea level change.

To further examine the potential productivity of clam gardens and what constitutes a true control, several experiments and surveys could be performed. We recommend sampling clam garden beach sediment cores to quantify pre-modification beach characteristics. Indeed, building experimental clam gardens or deconstructing them, while quantifying ecological and physical responses before and after the modification at both treatment and control sites would be the ultimate way to test the effects of clam gardens on clam productivity. The experimental modification of substrate (i.e. adding shell hash), elimination of clam predators (i.e. *Pycnopodia helianthoides* and *Pisastser brevispinus*), and reduction of density dependence (i.e. reducing the density of juvenile clams) could also help tease apart other detailed mechanisms driving increased productivity in clam gardens.

### Enhancing Food Security Confers Resilience to Social Ecological Systems

An increased appreciation of the coupling between ecosystems and human well-being has triggered a paradigm shift in the applied ecological sciences towards a focus on understanding the dynamics of coupled social-ecological systems (SES), linked systems of people and nature [55], [56], [23]. In many marine systems, current management approaches have demonstrably failed to halt or reverse fisheries declines [57], in part due to the inadequate recognition of the strong links between social and ecological processes [58], [59]. Ancient clam gardens and their governance by coastal communities are an example of an adaptive strategy that likely enhanced regional food security and thus conferred resilience to these coupled human-coastal ocean ecosystems.

Our observations on the variation in this ancient form of mariculture also highlight key aspects of resilient social-ecological-systems. The general uniformity in tidal height of clam garden walls on Quadra -- which likely date to different time periods and were owned and managed by different social groups -- reflects knowledge that was shared inter-generationally and across communities. As for variation, we suggest that the two clam gardens that fell outside the optimal tidal height range could be representations of engineering errors and learning, or perhaps, were built during times of differing sea levels. Alternatively, these features may have been built to target other clam species or may have had purposes other than shellfish harvest.

Food security is not only a contemporary issue. It has motivated ingenuity and development of civilizations throughout time. Investigations of how ancient clam gardens work will provide information on possible solutions to local food security and economic resiliency of coastal communities. Based on our clam surveys on Quadra Island, densities of *L. staminea* and *S. giganteus* within clam gardens are elevated on average by 151% and 300%, respectively, within clam gardens (Table S2). Clam garden biomass of *L. staminea* and *S. giganteus* were elevated on average by 68% and 253%, respectively (Table S2). Estimates made from our experiments indicate that clam gardens within optimal clam habitat can enhance growth rates of *L. staminea* on average by 89%, meaning that clams reach harvestable size at a faster rate (Table S2). If we had chosen to transplant *S. giganteus*, we predict that we would have detected higher growth rates in clam gardens compared to non-walled beaches, but isolated to the lower half of clam gardens, within *S. giganteus* optimal habitat. The archaeological record is clear; abundant shellfish have supported large populations of people on the Northwest Coast through history [60], [8]. This new evidence helps emphasize the value of incorporating traditional management techniques into future strategies towards sustainable solutions, contributing to local food security efforts globally.

### Informing Contemporary and Future Marine Management

Finding solutions to meet ecologically sound food production for the growing demands is a global effort, even though successful remedies may be locally adapted. Local food production is essential to community food security and autonomy [61]. Autonomous economies have been found to, out of necessity, recognize ecological limits, and protect biological, cultural and social diversity [62]. Some of today’s benthic shellfish aquaculture practices have been shown to alter the community composition of nearshore systems [63], change sediment characteristics [64], and facilitate the introduction of invasive species [65], [66]. Ecosystem impacts of modern harvest techniques that do not prioritize conservation of ecosystem biodiversity as well as productivity can undermine nearshore ecosystem resilience [67]. These are very real concerns for coastal First Nations. Documenting these traditional practices and their ecological and societal benefits will help First Nations during a pivotal time, as First Nations continue to assert their rights to access traditional lands and resources and secure sustainable food production into the future.

## Supporting Information

Figure S1 **Mean clam garden terrace intertidal height.** Mean Intertidal Height (+/– min and max terrace height) of eleven clam gardens in Waiatt Bay and Kanish Bay, British Columbia, Canada. n = 6. Dashed lines represent optimal tidal height for *L. staminea* in Kanish Bay (darker line, 0.7–1.9 m) and Waiatt Bay (lighter line, 0.6–1.6 m) as determined by our survey data of *L. staminea* density experimental growth rates of non-walled beaches (Fig. S4a,b).
(TIF)

Figure S2 **Site characteristics: Mean intertidal height and slope.** A) Mean intertidal height (m above LLWLT +/– SE) and B) slope (▵y/▵x +/–SE) across survey transects spanning from the top of clam habitat to top of clam garden wall within clam gardens, and to ∼0.75 m intertidal height within non-walled beaches in Waiatt and Kanish Bay, British Columbia, Canada.
(TIF)

Figure S3 **Effect of clam garden treatment on survival and growth.** A,C) Survival (+/–SE) and B,D) growth (+/–SE) of transplanted *L. staminea* (n = 15 individuals/outplant bag) over 160 days in clam gardens (n = 6) and non-walled beaches (n = 5). Note: D) includes gardens WB33,36,39,42 and excludes WB10 and WB31.
(TIF)

Figure S4 **Mean proportional growth of transplanted** ***L. staminea*** **.** A) Proportional growth (mean of site means +/–SE) of transplanted *L. staminea* over 160 days in Clam Gardens (n = 6 sites, n_site_ = 5) and Non-Walled Beaches (n = 5, n_site_ = 5) (F_(4,9)_ = 1.576, p = 0.241). and B) growth excluding gardens terraces at optimal tidal height extremes WB10 and WB31 (F_(4,7)_ = 11.947, p = 0.011*).
(TIF)

Table S1 **Site Characteristics.** Means and standard errors of all measured site characteristics by Site Type and Bay.
(PPTX)

Table S2 **Summary Table: Effect of clam garden treatment on all measured responses.** Means and standard errors for all measured response variables of bivalve productivity by site type.
(PPTX)

Table S3 **GLMMs Summary.** The effects of clam gardens (Beach Type) on experimentally transplanted *L. staminea* survivorship and growth. Analysis of GLMMs with Beach Type as a fixed effect (i.e. clam garden vs. non-walled beach) and Site as a random effect. * designates significant p-values (p≤0.05).
(PPTX)
