# Supplementary figures and images for: Ancient Clam Gardens Increased Shellfish Production: Adaptive Strategies from the Past Can Inform Food Security Today
Source: PLoS One. 2014 Mar 11;9(3):e91235. doi: 10.1371/journal.pone.0091235 (PMC3949788; doi:10.1371/journal.pone.0091235)

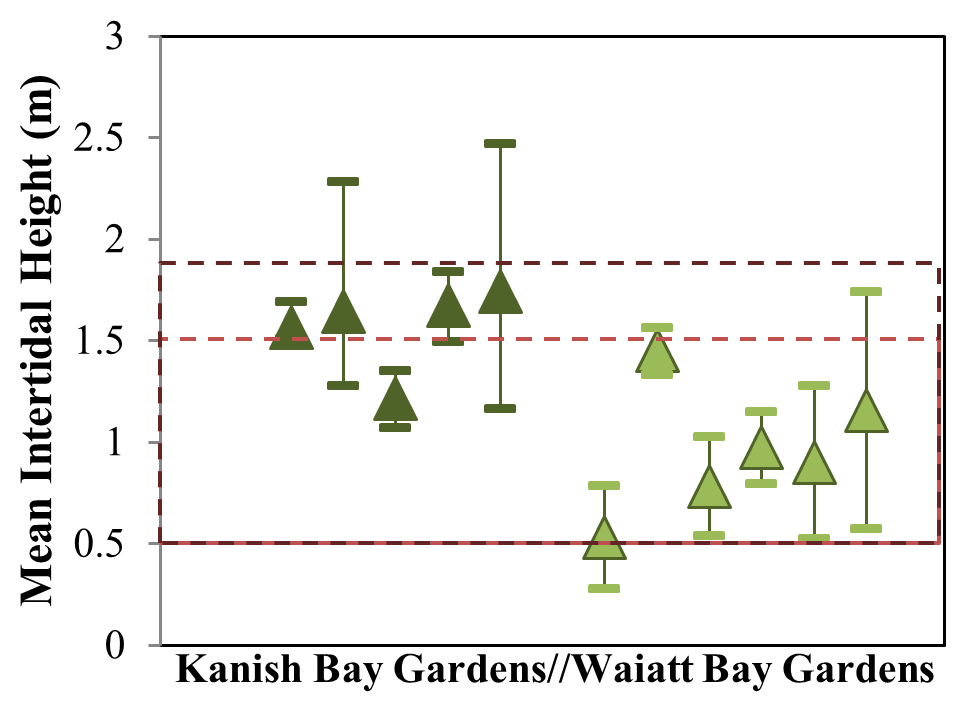

Supplement: Figure S1 — Mean clam garden terrace intertidal height. Mean Intertidal Height (+/– min and max terrace height) of eleven clam gardens in Waiatt Bay and Kanish Bay, British Columbia, Canada. n = 6. Dashed lines represent optimal tidal height for L. staminea in Kanish Bay (darker line, 0.7–1.9 m) and Waiatt Bay (lighter line, 0.6–1.6 m) as determined by our survey data of L. staminea density experimental growth rates of non-walled beaches (Fig. S4a,b). (TIF) [file pone.0091235.s001.tif]

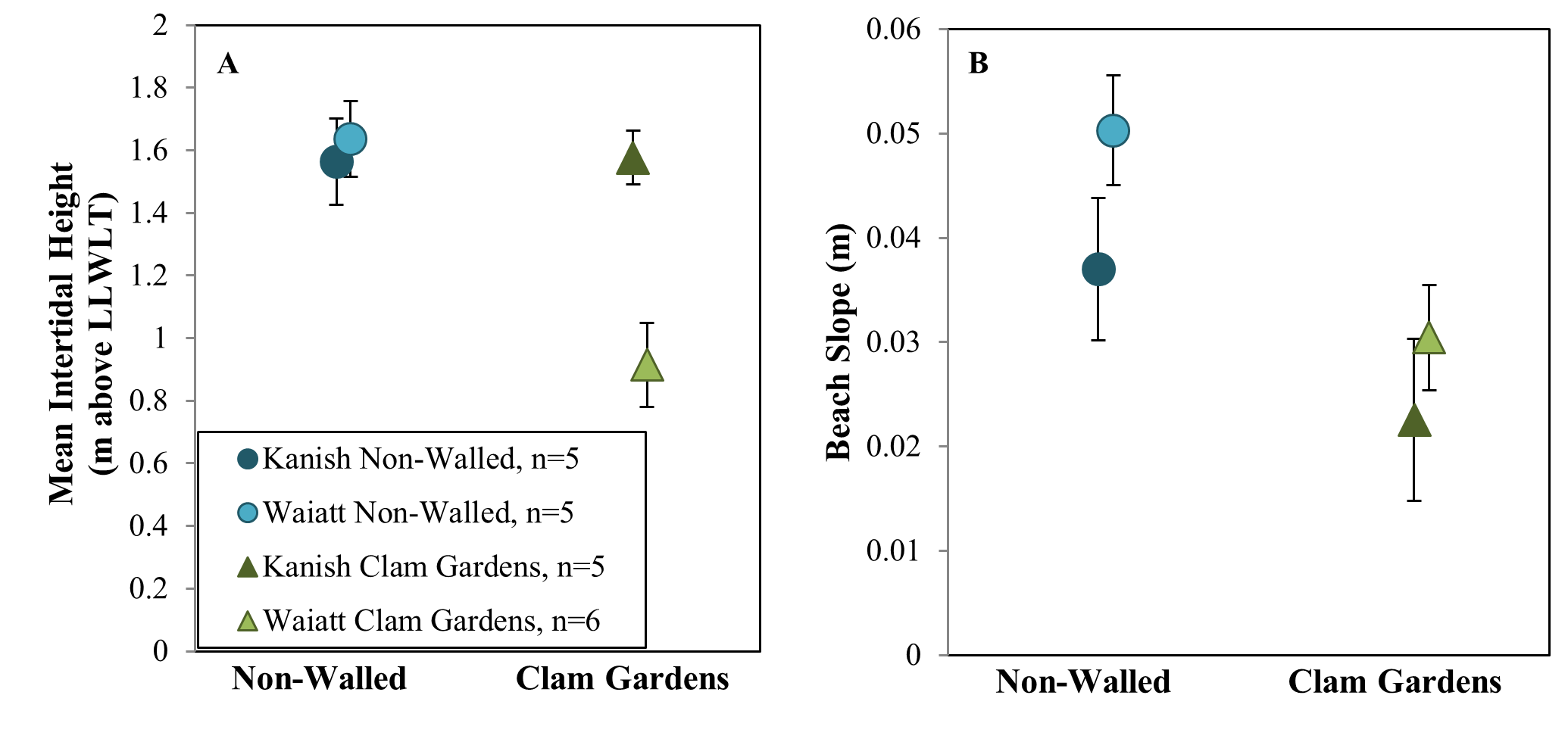

Supplement: Figure S2 — Site characteristics: Mean intertidal height and slope. A) Mean intertidal height (m above LLWLT +/– SE) and B) slope (▵y/▵x +/–SE) across survey transects spanning from the top of clam habitat to top of clam garden wall within clam gardens, and to ∼0.75 m intertidal height within non-walled beaches in Waiatt and Kanish Bay, British Columbia, Canada. (TIF) [file pone.0091235.s002.tif]

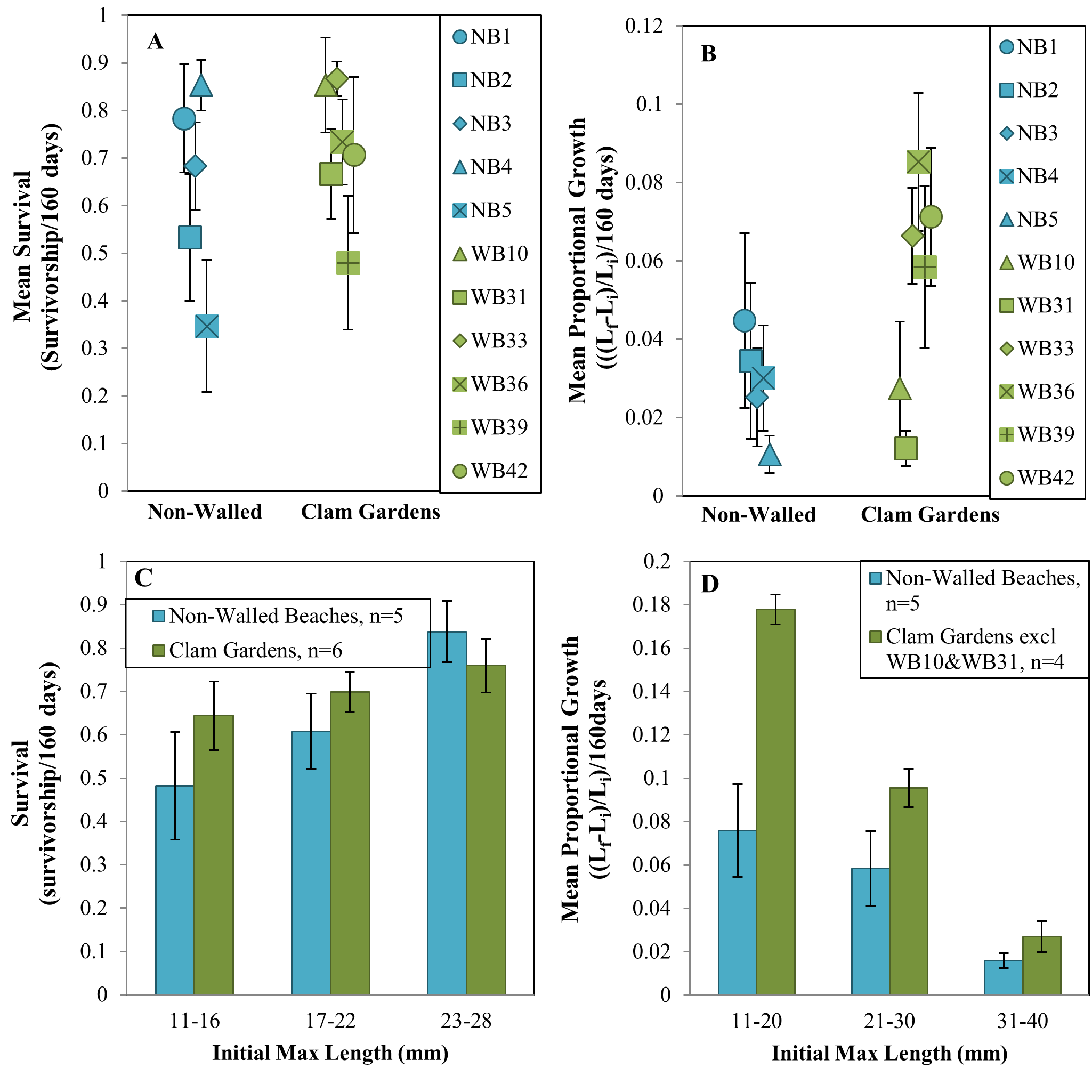

Supplement: Figure S3 — Effect of clam garden treatment on survival and growth. A,C) Survival (+/–SE) and B,D) growth (+/–SE) of transplanted L. staminea (n = 15 individuals/outplant bag) over 160 days in clam gardens (n = 6) and non-walled beaches (n = 5). Note: D) includes gardens WB33,36,39,42 and excludes WB10 and WB31. (TIF) [file pone.0091235.s003.tif]

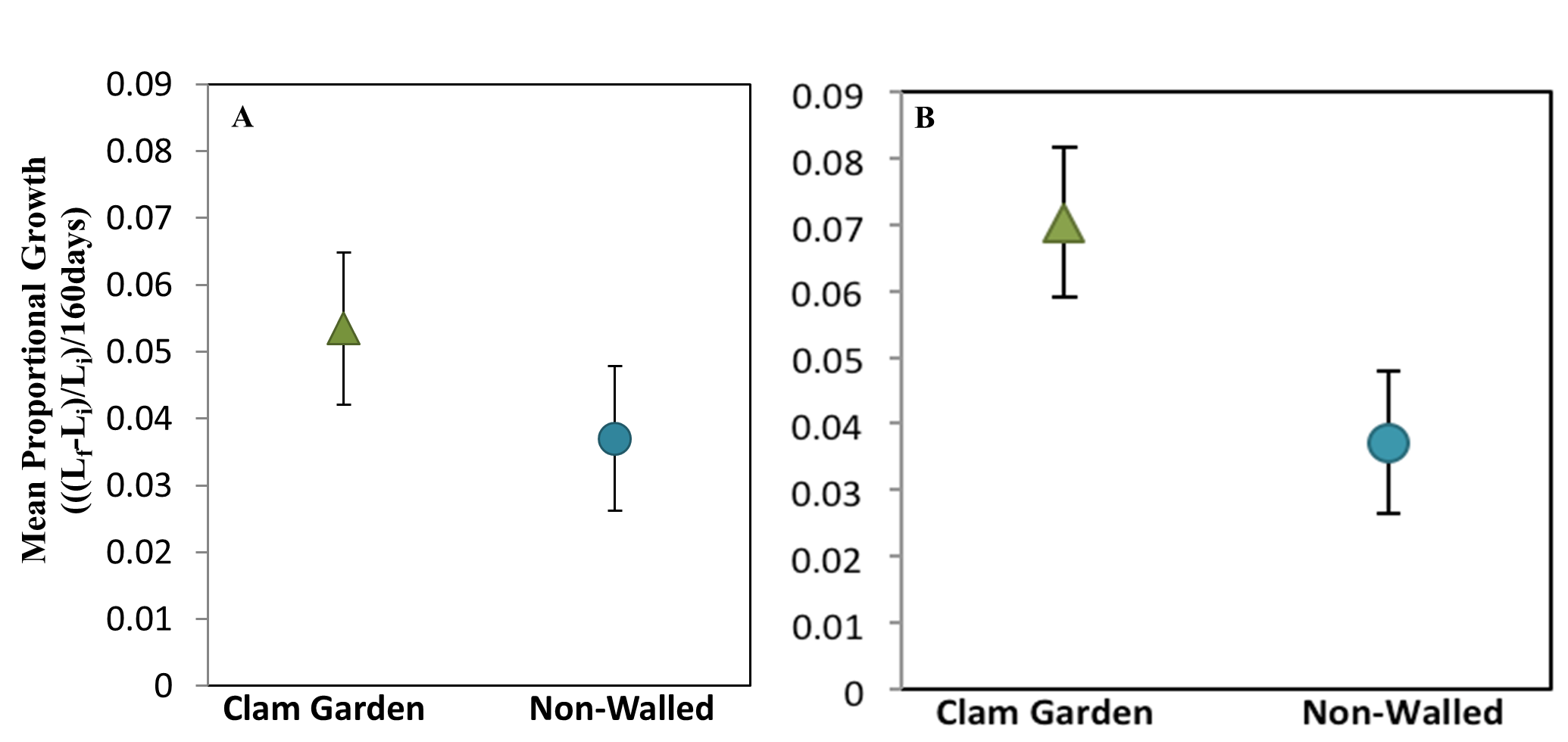

Supplement: Figure S4 — Mean proportional growth of transplanted L. staminea . A) Proportional growth (mean of site means +/–SE) of transplanted L. staminea over 160 days in Clam Gardens (n = 6 sites, nsite = 5) and Non-Walled Beaches (n = 5, nsite = 5) (F(4,9) = 1.576, p = 0.241). and B) growth excluding gardens terraces at optimal tidal height extremes WB10 and WB31 (F(4,7) = 11.947, p = 0.011*). (TIF) [file pone.0091235.s004.tif]
