# Supplementary material for: Ancient Clam Gardens Increased Shellfish Production: Adaptive Strategies from the Past Can Inform Food Security Today
Source: PLoS One. 2014 Mar 11;9(3):e91235. doi: 10.1371/journal.pone.0091235 (PMC3949788; doi:10.1371/journal.pone.0091235)
Supplement: Table S1 — Site Characteristics. Means and standard errors of all measured site characteristics by Site Type and Bay. (PPTX) [file pone.0091235.s005.pptx]

## Slide 1
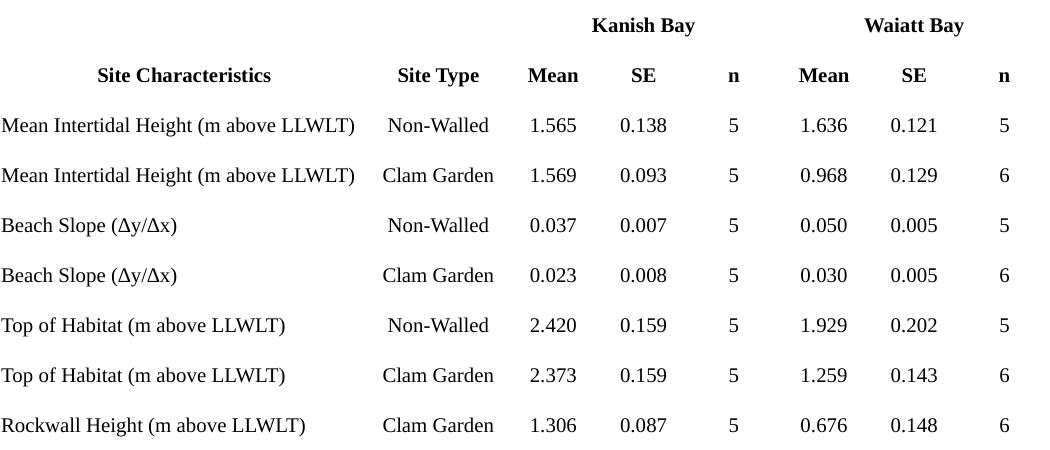

| | | Kanish Bay | | | Waiatt Bay | | |
| --- | --- | --- | --- | --- | --- | --- | --- |
| Site Characteristics | Site Type | Mean | SE | n | Mean | SE | n |
| Mean Intertidal Height (m above LLWLT) | Non-Walled | 1.565 | 0.138 | 5 | 1.636 | 0.121 | 5 |
| Mean Intertidal Height (m above LLWLT) | Clam Garden | 1.569 | 0.093 | 5 | 0.968 | 0.129 | 6 |
| Beach Slope (∆y/∆x) | Non-Walled | 0.037 | 0.007 | 5 | 0.050 | 0.005 | 5 |
| Beach Slope (∆y/∆x) | Clam Garden | 0.023 | 0.008 | 5 | 0.030 | 0.005 | 6 |
| Top of Habitat (m above LLWLT) | Non-Walled | 2.420 | 0.159 | 5 | 1.929 | 0.202 | 5 |
| Top of Habitat (m above LLWLT) | Clam Garden | 2.373 | 0.159 | 5 | 1.259 | 0.143 | 6 |
| Rockwall Height (m above LLWLT) | Clam Garden | 1.306 | 0.087 | 5 | 0.676 | 0.148 | 6 |
