# Supplementary material for: Ancient Clam Gardens Increased Shellfish Production: Adaptive Strategies from the Past Can Inform Food Security Today
Source: PLoS One. 2014 Mar 11;9(3):e91235. doi: 10.1371/journal.pone.0091235 (PMC3949788; doi:10.1371/journal.pone.0091235)
Supplement: Table S2 — Summary Table: Effect of clam garden treatment on all measured responses. Means and standard errors for all measured response variables of bivalve productivity by site type. (PPTX) [file pone.0091235.s006.pptx]

## Slide 1
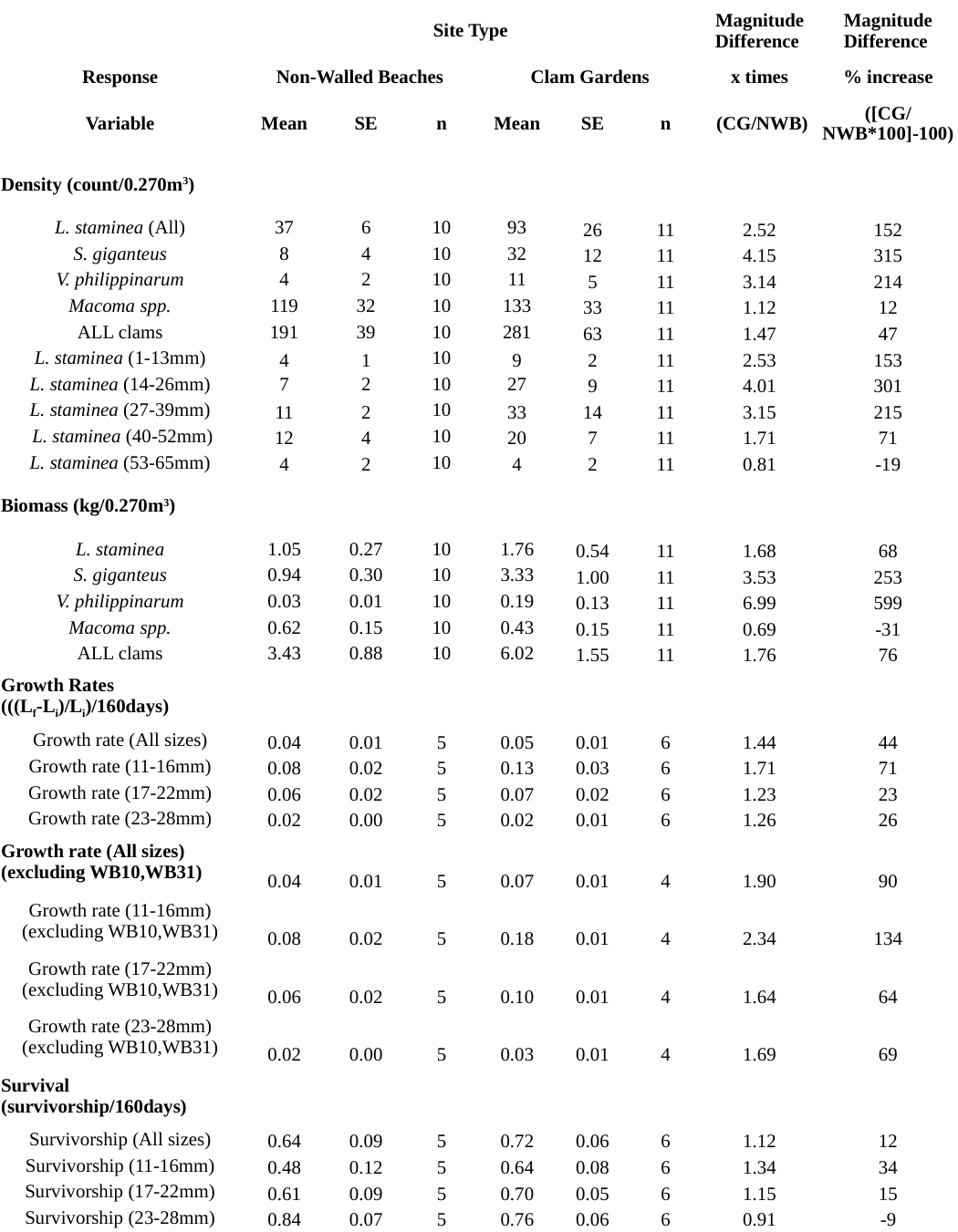

| | Site Type | | | | | | Magnitude Difference | Magnitude Difference |
| --- | --- | --- | --- | --- | --- | --- | --- | --- |
| Response | Non-Walled Beaches | | | Clam Gardens | | | x times | % increase |
| Variable | Mean | SE | n | Mean | SE | n | (CG/NWB) | ([CG/NWB\*100]-100) |
| Density (count/0.270m3) | | | | | | | | |
| L. staminea (All) | 37 | 6 | 10 | 93 | 26 | 11 | 2.52 | 152 |
| S. giganteus | 8 | 4 | 10 | 32 | 12 | 11 | 4.15 | 315 |
| V. philippinarum | 4 | 2 | 10 | 11 | 5 | 11 | 3.14 | 214 |
| Macoma spp. | 119 | 32 | 10 | 133 | 33 | 11 | 1.12 | 12 |
| ALL clams | 191 | 39 | 10 | 281 | 63 | 11 | 1.47 | 47 |
| L. staminea (1-13mm) | 4 | 1 | 10 | 9 | 2 | 11 | 2.53 | 153 |
| L. staminea (14-26mm) | 7 | 2 | 10 | 27 | 9 | 11 | 4.01 | 301 |
| L. staminea (27-39mm) | 11 | 2 | 10 | 33 | 14 | 11 | 3.15 | 215 |
| L. staminea (40-52mm) | 12 | 4 | 10 | 20 | 7 | 11 | 1.71 | 71 |
| L. staminea (53-65mm) | 4 | 2 | 10 | 4 | 2 | 11 | 0.81 | -19 |
| Biomass (kg/0.270m3) | | | | | | | | |
| L. staminea | 1.05 | 0.27 | 10 | 1.76 | 0.54 | 11 | 1.68 | 68 |
| S. giganteus | 0.94 | 0.30 | 10 | 3.33 | 1.00 | 11 | 3.53 | 253 |
| V. philippinarum | 0.03 | 0.01 | 10 | 0.19 | 0.13 | 11 | 6.99 | 599 |
| Macoma spp. | 0.62 | 0.15 | 10 | 0.43 | 0.15 | 11 | 0.69 | -31 |
| ALL clams | 3.43 | 0.88 | 10 | 6.02 | 1.55 | 11 | 1.76 | 76 |
| Growth Rates (((Lf-Li)/Li)/160days) | | | | | | | | |
| Growth rate (All sizes) | 0.04 | 0.01 | 5 | 0.05 | 0.01 | 6 | 1.44 | 44 |
| Growth rate (11-16mm) | 0.08 | 0.02 | 5 | 0.13 | 0.03 | 6 | 1.71 | 71 |
| Growth rate (17-22mm) | 0.06 | 0.02 | 5 | 0.07 | 0.02 | 6 | 1.23 | 23 |
| Growth rate (23-28mm) | 0.02 | 0.00 | 5 | 0.02 | 0.01 | 6 | 1.26 | 26 |
| Growth rate (All sizes) (excluding WB10,WB31) | 0.04 | 0.01 | 5 | 0.07 | 0.01 | 4 | 1.90 | 90 |
| Growth rate (11-16mm) (excluding WB10,WB31) | 0.08 | 0.02 | 5 | 0.18 | 0.01 | 4 | 2.34 | 134 |
| Growth rate (17-22mm) (excluding WB10,WB31) | 0.06 | 0.02 | 5 | 0.10 | 0.01 | 4 | 1.64 | 64 |
| Growth rate (23-28mm) (excluding WB10,WB31) | 0.02 | 0.00 | 5 | 0.03 | 0.01 | 4 | 1.69 | 69 |
| Survival (survivorship/160days) | | | | | | | | |
| Survivorship (All sizes) | 0.64 | 0.09 | 5 | 0.72 | 0.06 | 6 | 1.12 | 12 |
| Survivorship (11-16mm) | 0.48 | 0.12 | 5 | 0.64 | 0.08 | 6 | 1.34 | 34 |
| Survivorship (17-22mm) | 0.61 | 0.09 | 5 | 0.70 | 0.05 | 6 | 1.15 | 15 |
| Survivorship (23-28mm) | 0.84 | 0.07 | 5 | 0.76 | 0.06 | 6 | 0.91 | -9 |
