# Supplementary material for: Ancient Clam Gardens Increased Shellfish Production: Adaptive Strategies from the Past Can Inform Food Security Today
Source: PLoS One. 2014 Mar 11;9(3):e91235. doi: 10.1371/journal.pone.0091235 (PMC3949788; doi:10.1371/journal.pone.0091235)
Supplement: Table S3 — GLMMs Summary. The effects of clam gardens (Beach Type) on experimentally transplanted L. staminea survivorship and growth. Analysis of GLMMs with Beach Type as a fixed effect (i.e. clam garden vs. non-walled beach) and Site as a random effect. * designates significant p-values (p≤0.05). (PPTX) [file pone.0091235.s007.pptx]

## Slide 1
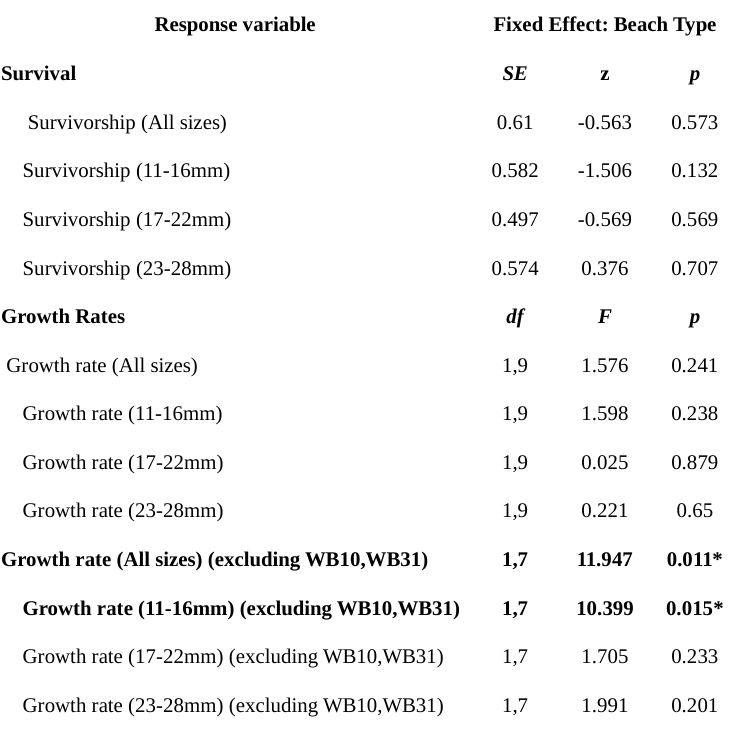

| Response variable | Fixed Effect: Beach Type | | |
| --- | --- | --- | --- |
| Survival | SE | z | p |
| Survivorship (All sizes) | 0.61 | -0.563 | 0.573 |
| Survivorship (11-16mm) | 0.582 | -1.506 | 0.132 |
| Survivorship (17-22mm) | 0.497 | -0.569 | 0.569 |
| Survivorship (23-28mm) | 0.574 | 0.376 | 0.707 |
| Growth Rates | df | F | p |
| Growth rate (All sizes) | 1,9 | 1.576 | 0.241 |
| Growth rate (11-16mm) | 1,9 | 1.598 | 0.238 |
| Growth rate (17-22mm) | 1,9 | 0.025 | 0.879 |
| Growth rate (23-28mm) | 1,9 | 0.221 | 0.65 |
| Growth rate (All sizes) (excluding WB10,WB31) | 1,7 | 11.947 | 0.011\* |
| Growth rate (11-16mm) (excluding WB10,WB31) | 1,7 | 10.399 | 0.015\* |
| Growth rate (17-22mm) (excluding WB10,WB31) | 1,7 | 1.705 | 0.233 |
| Growth rate (23-28mm) (excluding WB10,WB31) | 1,7 | 1.991 | 0.201 |
